# TRPC6 effects on albumin permeation, nephrin shedding, and apoptosis in podocytes: Role of calcineurin and metalloproteases

**DOI:** 10.14814/phy2.70614

**Published:** 2025-10-30

**Authors:** Eun Young Kim, Stuart E. Dryer

**Affiliations:** ^1^ Department of Biology and Biochemistry University of Houston Houston Texas USA; ^2^ Department of Biomedical Sciences, Tilman J. Fertitta Family College of Medicine University of Houston Houston Texas USA

**Keywords:** apoptosis, cation channel, effacement, nephrin shedding, podocyte

## Abstract

The effects of TRPC6 activation for various periods of time on three classes of functional outputs were examined in cultured podocytes: albumin permeation across a confluent layer; changes in nephrin dynamics; and cell death. Albumin permeation in transwell assays was significantly increased within 1 h in response to the activation of formyl peptide receptors (FPR), but the TRPC6 inhibitor SAR‐7334 had no effect on this response, and 1 h or 24 h exposures to the TRPC6 activator PPZ2 did not increase albumin permeation. Direct TRPC6 activation for 24 h evoked an increase in shedding of nephrin ectodomains into the surrounding media, accompanied by an increase in matrix metalloprotease‐7 (MMP‐7). These effects were blocked by the calcineurin inhibitor cyclosporin A (CsA), as well as by Batimastat, a broad‐spectrum inhibitor of metalloproteinases including MMP‐7. TRPC6 activation for 24 h also evoked an increase in occludin abundance but had no effect on the abundance of podocin. Finally, TRPC6 activation for 72 h, but not for 24 h, evoked an increase in apoptotic cell death based on increases in cleaved caspase‐3. This effect was blocked by both SAR‐7334 and CsA. TRPC6 activation did not induce pyroptosis based on the measurement of cleaved gasdermin D.

## Graphical abstract

Certain ligands of pattern recognition receptors can induce a rapid increase in albumin permeability across a podocyte monolayer that can be seen in 1 h, but activation of TRPC6 for up to 24 h does not mimick this process. However, continuous TRPC6 activation for 24 h results in nephrin ectodomain shedding that calcineurin activation and metalloproteinases and that is is accompanied by an increase in overall abundance of occludin and matrix metalloprotreinase‐7. Finally, continuous TRPC6 activation for 72 h induces apoptosis as reported earlier, but does not induce pyroptosis.

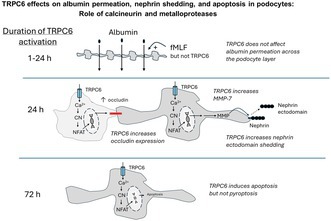

## 1 INTRODUCTION

Abnormal function of canonical transient receptor potential‐6 channels (TRPC6) has been implicated in the pathogenesis of familial and acquired forms of severe nephrotic syndromes (Staruschenko et al., 2023). A number of gain‐of‐function mutations in TRPC6 result in autosomal dominant forms of focal segmental glomerulosclerosis (FSGS) (Heeringa et al., 2009; Reiser et al., 2005; Winn et al., 2005), and an increased abundance of glomerular TRPC6 proteins has been observed in patients with primary FSGS (Möller et al., 2009) and in animal models of glomerular disease (Kim et al., 2018; Staruschenko et al., 2023). Certain TRPC6 mutations that cause a complete loss of cell surface cation channel function have also been associated with early onset FSGS (Riehle et al., 2016), possibly through mistargeting of the channel proteins or by affecting non‐channel functions of these proteins (Farmer et al., 2019; Riazanski et al., 2015). However, mice and rats in which TRPC6 is knocked out or completely inactivated show no signs of kidney dysfunction and are at least partially protected in several kidney disease models (Dryer & Kim, 2022; Kim et al., 2018; Staruschenko et al., 2023). Supported by an extensive preclinical literature (Dryer & Kim, 2022; Staruschenko et al., 2023), phase 2 clinical trials of a recently developed TRPC6 inhibitor for the treatment of chronic kidney disease are now underway (Trachtman et al., 2023).

Notwithstanding their implication in certain kidney diseases, it is likely that TRPC6 channels have normal physiological functions in podocytes, as they are components of rapidly acting GPCR pathways tied to Ca^2+^ influx in podocytes, including signals mediated by angiotensin II (Ilatovskaya et al., 2014), P2Y receptors (Roshanravan & Dryer, 2014), κ‐opiate receptors (Golosova et al., 2020), bioactive lipids (Roshanravan et al., 2016), and metabotropic glutamate receptors (Wang et al., 2019). Podocyte TRPC6 channels also become active in response to mechanical stimuli (Anderson et al., 2013; Gyarmati et al., 2021) and their activation can be enhanced following treatment with immunomodulatory signals that act over a longer time course, such as following exposure to the soluble urokinase plasminogen activator receptor (suPAR) or TNFα (Kim et al., 2017).

In the present study, we have examined the role of TRPC6 channels in mediating three distinct classes of cellular processes in cultured podocytes: (1) albumin movement across a confluent layer of podocytes; (2) changes in proteins that are components of slit diaphragm and occluding junctions, with a special focus on nephrin; and (3) cell death. These experiments were carried out in vitro to avoid factors originating in other cell types that complicate the interpretation of experiments in which various agents such as lipopolysaccharides (LPS), TNF, or suPAR are injected or overexpressed in vivo. We have also taken advantage of relatively recently developed pharmacological tools that allow for direct activation (Sawamura et al., 2016) and inhibition (Maier et al., 2015) of TRPC6 channels. Here we report that TRPC6 activation for 1–24 h has no effect on the permeability of a confluent monolayer of podocytes to albumin, but nephrin shedding and increased expression of occludin occur after 24 h. The shedding of nephrin ectodomains required activation of calcineurin‐dependent signaling cascades and metalloproteinases. As expected from many earlier papers (Staruschenko et al., 2023), more sustained TRPC6 activation (72 h) triggered apoptosis. However, this treatment did not appear to induce pyroptosis.

## 2 MATERIALS AND METHODS

### 2.1 Cell culture

All experiments utilized an immortalized mouse podocyte cell line (MPC‐5 cells) obtained from Dr. Peter Mundel. The passage number of the cells used in these experiments was <15. Cells were propagated at 33°C at a low density, and terminal differentiation into a podocyte phenotype was induced by the removal of γ‐interferon and a temperature switch to 37°C for 14 days, as described previously (Kim et al., 2008). We periodically confirm that podocytes cultured in this way express nephrin and podocin after differentiation.

### 2.2 Albumin flux assays

**FIGURE 1:**
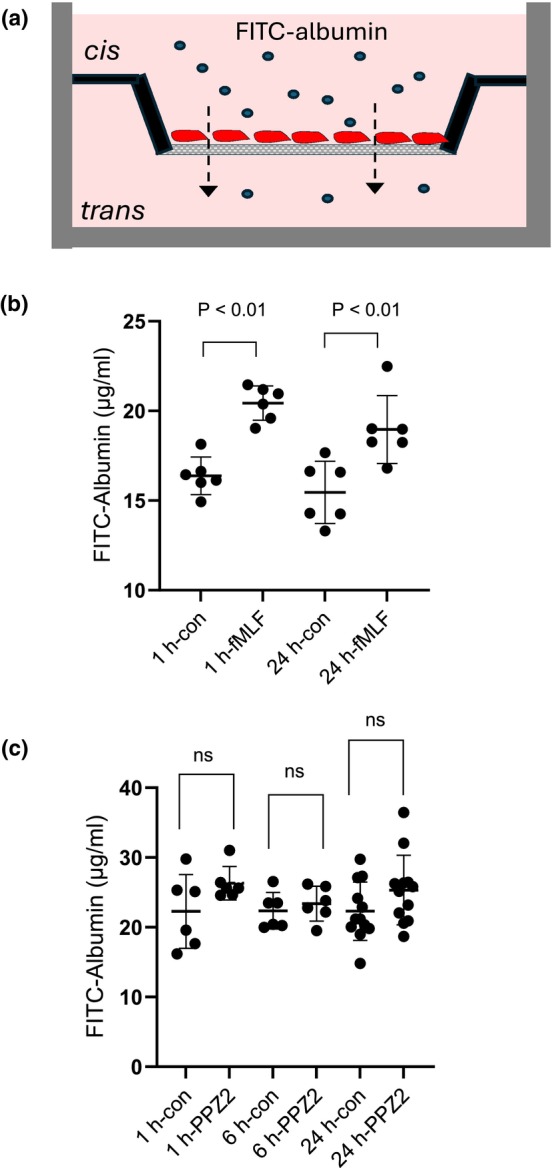
Albumin permeation across a podocyte monolayer. (a) A schematic diagram of the transwell assays used to assess albumin permeability. Following various treatments, FITC‐albumin is introduced into the cell culture media on the *cis* side of the transwell chamber. A confluent layer of differentiated MPC‐5 podocytes is growing on the transwell insert. After a period of time to allow for permeation, FITC‐albumin is quantified in the fluid in the *trans* side of the chamber. (b) The albumin permeation assay was carried out after cells were treated for 1 or 24 h with normal media or media containing 1 μM fMLF, an agonist of formyl peptide receptors. Exposure to fMLF evoked a significant increase in albumin permeation across the podocyte layer (Student's unpaired *t*‐test). Note that the data in this experiment reflect albumin permeation over a period of 1 h following fMLF treatment. In this and subsequent graphs, points show results of individual chambers along and bars denote mean ± SD. (c) Exposing podocytes to the TRPC6 activator PPZ2 (10 μM) for 1 h, 6 h, or 24 h had no effect on albumin permeation across the podocyte layer. Data in this graph reflect net albumin permeation over a period of 3 h following PPZ2 treatment.

The experimental approach used here is similar to that of Hunt et al. (2005), who demonstrated that MPC‐5 cells form size‐selective barriers with a selectivity similar to that seen in glomerular filtration barriers in vivo. For these experiments, a confluent layer of MPC‐5 precursor cells was plated onto the inserts of 12 mm Corning Transwell™ plates (0.4 μM pore size, catalog number 3460) and then differentiated into a podocyte phenotype. Cells were subjected to various treatments as described in detail further below. Drugs were applied in normal cell culture medium and cells were maintained at 37° in a CO_2_ incubator. At the end of the various treatments, fluorescein isothiocyanate‐tagged albumin (FITC‐albumin) obtained from Millipore Sigma (St. Louis, MO, USA, catalog number A9771) and dissolved in normal culture media at a concentration of 1 mg/mL was introduced onto the *cis* side of the Transwell chamber. In most of the experiments, cells were returned to the CO_2_ incubator for 3 h (Figure 1a). In a series of pilot experiments, we observed that this time course allowed for reproducible albumin permeation measurements. At that time, media on the *trans* sides of the chambers were then collected, and FITC‐albumin was quantified by measuring fluorescence (excitation at 490 nm and emission at 530 nm). We note, however, that in our earliest experiments, which include the data shown in Figure 1b, the time allowed for albumin permeation was only 1 h.

### 2.3 Immunoblot and ELISA analyses

In these experiments, podocytes were grown on conventional cell culture dishes. Proteins in podocyte lysates were separated on 10% SDS‐PAGE gels and then transferred to membranes. The blots were incubated with a primary antibody overnight at 4°C, followed by horseradish peroxidase (HRP)‐conjugated secondary antibody for 1 h at room temperature. Antibodies against cleaved caspase‐3 (catalog number 9661S) and cleaved Gasdermin D (catalog number 36425S) were obtained from Cell Signaling Inc. (Danvers, MA, USA). Antibodies against occludin (sc‐133256) and podocin (sc‐518088) were obtained from Santa Cruz Biotechnology (Santa Cruz, CA, USA). An antibody against MMP‐7 (catalog number 10374‐2‐AP) was obtained from Proteintech Group Inc. (Rosemont, IL, USA). These antibodies were used at a dilution of 1:1000. In addition, an antibody against actin was obtained from Millipore Sigma (catalog number ZMS1004) and was used at a dilution of 1:5000. Secondary antibodies used to probe blots were HRP‐linked anti‐rabbit IgG Antibody (catalog number 7074) and HRP‐linked anti‐mouse IgG (catalog number 7076), both from Cell Signaling Technology Inc. and both used at a dilution of 1:5000. Proteins were visualized using a SuperSignal™ West Pico PLUS Chemiluminescent Substrate (Thermo Fisher Inc., catalog number 34580) and signal intensities were quantified using Image J (Bethesda, MD, USA). Soluble nephrin present in culture media after various treatments was measured using the Mouse Nephrin ELISA kit (catalog number NBP2‐76750, Novus Biologicals, Centennial, CO, USA). Note that the media were centrifuged for 5 min at 3000 rpm to remove any detached cells or cellular debris prior to assays of the media.

### 2.4 Drugs

The formyl peptide receptor (FPR) agonist *N*‐Formyl‐Met‐Leu‐Phe (fMLF) was obtained from Tocris Bioscience (Minneapolis, MN, USA, catalog number 1921). The TRPC6 activator 2‐[4‐(2,3‐dimethylphenyl)piperazin‐1‐yl]‐*N*‐(2‐ethoxyphenyl)acetamide (PPZ2, catalog number 1596) (Sawamura et al., 2016) and cyclosporin A (CsA, catalog number 30024) were purchased from MilliporeSigma. The TRPC6 inhibitor (4‐[[(1*R*,2*R*)‐2‐[(3*R*)‐3‐amino‐1‐piperidinyl]‐2,3‐dihydro‐1*H*‐inden‐1‐yl]oxy]‐3‐chlorobenzonitrile dihydrochloride (SAR7334)) (Maier et al., 2015) was obtained from Cayman Chemical (Ann Arbor, MI, USA, catalog number 28292). Batimastat (BB‐94) was purchased from Medchem Express (Monmouth Junction, NJ, USA, catalog number HY‐13564).

### 2.5 Statistical analysis

Data were analyzed by Student's unpaired *t*‐test, or by one‐way ANOVA followed by Tukey's Honest Significant Difference post hoc test using Graphpad Prism v10 software. Graphs show results of independent measurements as points, and error bars denote ±SD.

## 3 RESULTS

### 3.1 TRPC6 activation does not affect albumin permeation across a podocyte monolayer

MPC‐5 podocytes form size‐selective barriers based on earlier studies using transwell assays similar to those used here (Hunt et al., 2005) and also form junctions with ultrastructural features similar to slit diaphragms that occur in vivo (Reiser et al., 2000). We have observed that increases in FITC‐albumin flux across the podocyte monolayer can occur relatively quickly after activation of certain signal transduction pathways. For example, this occurs after exposing podocytes to the formyl peptide receptor (FPR) agonist fMLF (1 μM) for 1 h or 24 h (Figure 1b). FPRs are a family of G protein coupled receptors that respond to certain damage‐ and pathogen‐associated molecular patterns, and whose activation induces increases in cytosolic ROS accumulation in cultured podocytes (Kim & Dryer, 2024). Given that cytosolic ROS can trigger TRPC6 mobilization and activation in podocytes (Staruschenko et al., 2023), we hypothesized that agents that cause direct TRPC6 activation would drive similar changes in albumin flux. However, in contrast to the prediction of that hypothesis, application of 10 μM PPZ2, a compound that causes direct activation of TRPC6 (Sawamura et al., 2016), had no discernible effect on albumin flux when it was applied for either 1, 6, or 24 h (Figure 1c). In addition, we observed that the TRPC6 blocker SAR‐7334 (1 μM) (Maier et al., 2015) had no effect on changes in albumin permeation evoked by 1 μM fMLF applied for 1 or 24 h (Figure S1).

### 3.2 TRPC6 activation induces matrix metalloproteinase‐mediated nephrin ectodomain shedding and upregulates occludin

**FIGURE 2:**
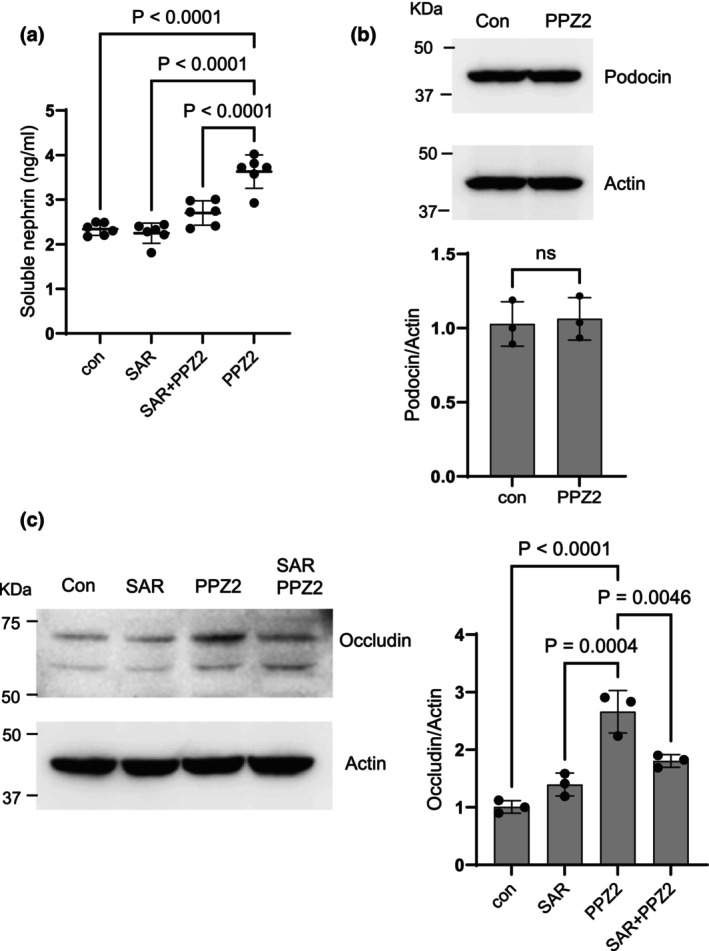
Effects of TRPC6 activation on junctional proteins of podocytes. (a) Treatment with the TRPC6 activator PPZ2 (10 μM) for 24 h induced an increase in soluble nephrin detected in surrounding cell culture media. This effect was blocked by concurrent exposure to the TRPC6 inhibitor SAR‐7334 (1 μM). Data were analyzed by one‐way ANOVA followed by Tukey's honest significant difference post hoc test. (b) TRPC6 activation by PPZ2 had no effect on the abundance of podocin as measured by immunoblot. A representative blot is shown above densitometric analyses of three repetitions of this experiment. Data were analyzed by Student's unpaired *t*‐test. (c) PPZ2 treatment for 24 h caused a significant increase in the abundance of occludin, and this effect was blocked by concurrent exposure to SAR‐7334. A representative immunoblot is shown to the left of densitometric analyses, and data were analyzed by one‐way ANOVA followed by Tukey's post hoc test.

We next examined if direct activation of TRPC6 would alter the expression or dynamics of proteins involved in the formation of specialized junctions in podocytes. We observed that the application of PPZ2 for 24 h resulted in a significant increase in soluble nephrin measured in the culture medium by ELISA (Figure 2a). This effect was completely blocked by concurrent exposure to the TRPC6 inhibitor SAR‐7334. Note that any cells that might have been present in the media were removed by centrifugation prior to the assay, and therefore this signal is unlikely to arise from detached podocytes. The application of PPZ2 for 24 h did not affect total podocin as measured by immunoblot (Figure 2b) but evoked a marked increase in the abundance of occludin. This later effect was also blocked by concurrent application of SAR‐7334 (Figure 2c).

**FIGURE 3:**
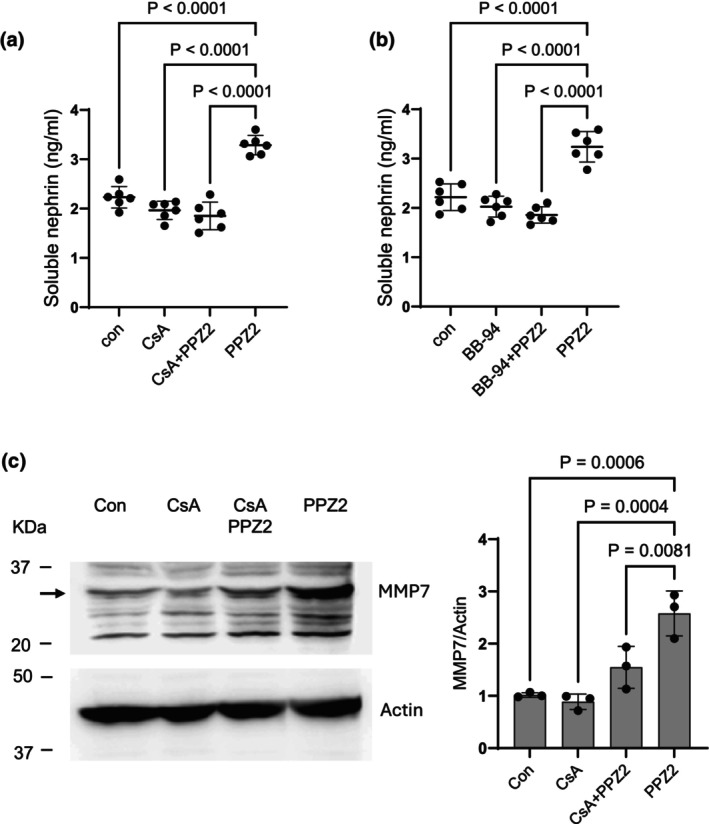
TRPC6 activation induces nephrin shedding mediated by calcineurin and metalloproteinases. (a) PPZ2 treatment for 24 h induces release of soluble nephrin, and this effect is inhibited by concurrent exposure to the calcineurin inhibitor CsA (1 μM). (b) PPZ2‐induced release of soluble nephrin is also inhibited by concurrent exposure to Batimastat (BB‐94), a broad‐spectrum inhibitor of matrix metalloproteinases. (c) PPZ2 treatment for 24 h caused a significant increase in the abundance of matrix metalloproteinase‐7 (MMP‐7), and this effect was blocked by concurrent exposure to CsA. Data were analyzed by one‐way ANOVA followed by Tukey's post hoc test.

We next examined some of the mechanisms whereby TRPC6 activation induces release of nephrin into the surrounding culture medium. The calcineurin‐NFAT pathway is one of the best characterized downstream pathways engaged following TRPC6 activation in podocytes (Nijenhuis et al., 2011; Schlöndorff et al., 2009). To test if this pathway is involved in nephrin release, we treated podocytes with PPZ2 for 24 h in the presence or absence of 1 μM CsA, a canonical inhibitor of calcineurin. We observed that CsA inhibited PPZ2‐evoked release of nephrin into the surrounding medium, but by itself had no effect (Figure 3a). Activated NFAT enters the nucleus and subsequently causes significant changes in patterns of gene expression, including activation of genes encoding matrix metalloproteases such as MMP‐3, MMP‐7, and MMP‐9 (Neria et al., 2013; Tie et al., 2013; Wang et al., 2015). This is notable because MMP‐7 has been shown to cleave nephrin ectodomains (Liu et al., 2020; Tan et al., 2019) and is upregulated in several glomerular diseases (Liu et al., 2020). Therefore, we examined if Batimastat (BB‐94), a broad‐spectrum inhibitor of matrix metalloproteinases including MMP‐7 and classic sheddases such as ADAM10 (Wojtowicz‐Praga et al., 1997), would affect nephrin release evoked by PPZ2. As with CsA, we observed that BB‐94 caused a complete inhibition of PPZ2 effects on nephrin release into the medium (Figure 3b). In addition, we observed that PPZ2 for 24 h also induced an increase in MMP‐7 expression in podocytes as measured by immunoblot, and this effect was inhibited by concurrent exposure to CsA, indicating that metalloproteinases lie downstream of the TRPC6‐calcineurin pathway in podocytes (Figure 3c).

### 3.3 Sustained TRPC6 activation induces apoptosis but not pyroptosis

**FIGURE 4:**
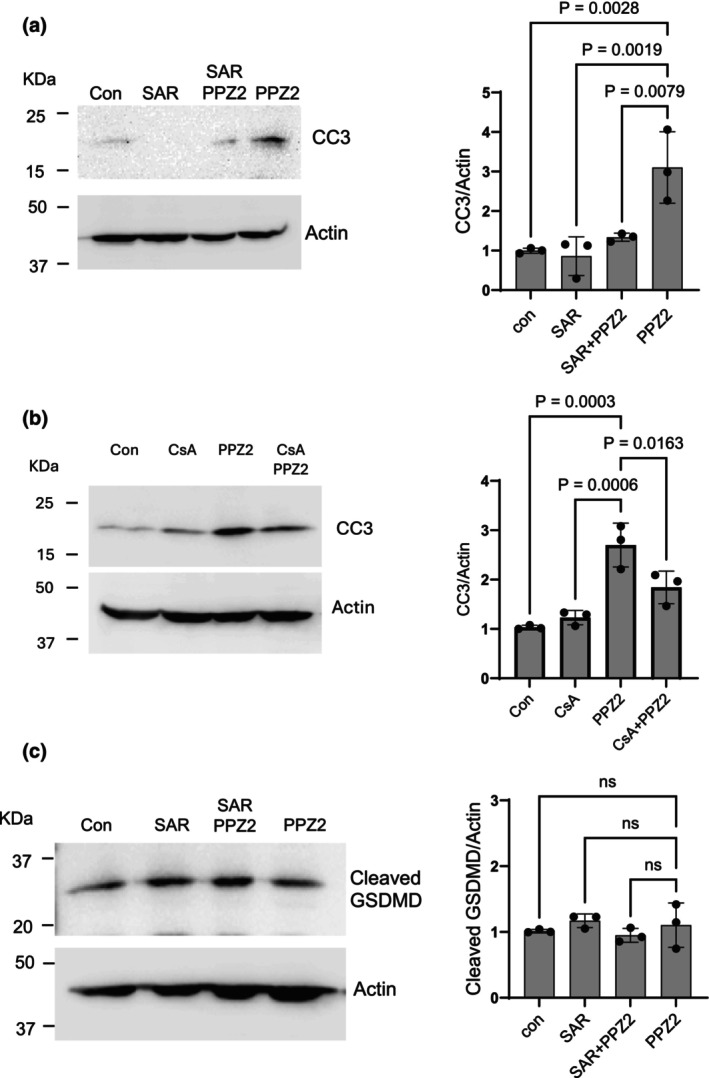
Continuous TRPC6 activation for 72 h induces apoptosis in podocytes. (a) Immunoblot analysis shows cleaved caspase‐3 (CC3), a marker for apoptosis, was increased in cells treated with PPZ2, and this effect was blocked by concurrent exposure to SAR‐7334. A representative blot is shown to the left, and a graph showing densitometric analysis of three repetitions of the experiment is shown to the right. (b) Effects of PPZ2 on CC3 were inhibited by concurrent exposure to CsA. (c) PPZ2 treatment for 72 h did not induce pyroptosis based on immunoblot analysis of cleaved gasdermin‐D (GSDMD). Data were analyzed by one‐way ANOVA followed by Tukey's post hoc test.

In these experiments we examined if PPZ2 can activate processes leading to cell death in podocytes. Based on substantial literature showing that TRPC6 channels can drive apoptosis (Staruschenko et al., 2023; Zhang et al., 2009), we examined processes driving various forms of cell death following 24 h and 72 h of continuous exposure to PPZ2 by measuring the abundance of cleaved caspase‐3 (CC3). PPZ2 had no effect on CC3 following a 24 h application (Figure S1). However, PPZ2 treatment for 72 h induced marked increases in CC3 that were completely blocked by concurrent exposure to SAR‐7334 (Figure 4a). This effect was also inhibited in cells concurrently treated with CsA (Figure 4b), which is consistent with earlier studies (Staruschenko et al., 2023; Zhang et al., 2009). We also examined the effects of PPZ2 on the abundance of cleaved gasdermin D, a marker for pyroptosis (Shi et al., 2017). In contrast to apoptosis markers, we did not observe any change in cleaved gasdermin D following a 72‐h exposure to PPZ2 (Figure 4c).

## 4 DISCUSSION

It is generally believed that periodic TRPC6 activation has a role in the normal function of podocytes (Gyarmati et al., 2021) but can become pathogenic if the activation is excessive in average duration and/or amplitude (Staruschenko et al., 2023). In the present study, we have examined the consequences of TRPC6 activation in a confluent layer of cultured podocytes, focusing on permeability to albumin, the behavior of certain important junctional proteins, and on processes that lead to podocyte cell death.

We were particularly interested in whether TRPC6 activation could regulate albumin flux across a confluent layer of podocytes in vitro. This hypothesis was motivated by multiple reports that transient and fully reversible proteinuria can occur in the absence of overt kidney disease, for example, during acute respiratory infections (Cho et al., 2013; Vehaskari & Rapola, 1982), and that this also occurs in mice following injection of pathogen‐associated molecular patterns such as lipopolysaccharide (Lorenzen et al., 2008; Reiser et al., 2004). Early on, we observed that activation of the FPR by its agonist fMLF can increase albumin permeability of a podocyte bilayer in as little as 1 h. This effect persisted during a continuous 24‐h exposure to fMLF, a time at which the transduction cascade switches to become β‐arrestin‐dependent as opposed to G protein‐dependent (Kim & Dryer, 2024). Given that FPRs are G protein‐coupled receptors that can activate Ca^2+^ signaling pathways (Yi et al., 2024), we predicted that a TRPC6‐activating small molecule would also alter albumin flux in this experimental system.

In contrast to our prediction, PPZ2 had no effect on FITC‐albumin flux when applied continuously for 15 min (not shown), 1 h, or 24 h. The MPC‐5 podocyte cell line used in these studies has previously been shown to form junctions with size‐selective permeation properties and cell junctions similar to those observed in vivo (Hunt et al., 2005; Reiser et al., 2000). These observations certainly do not exclude a role for podocyte TRPC6 channels in the acute regulation of glomerular filtration rate, for example during tubuloglomerular feedback (Gyarmati et al., 2021), but they argue against a role for TRPC6 in mediating transient changes in protein permeability that might occur, for example, as a result of activation of pattern recognition receptors (Myette et al., 2025).

By contrast, TRPC6 activation for 24 h caused marked changes in certain junctional proteins expressed in podocytes, including a marked increase in soluble nephrin in the surrounding culture media, that is, nephrin that remains after centrifugal pelleting of intact cells or cellular debris. Nephrin can be detected in the urine of humans and animals in the early stages of glomerular diseases (Mesfine et al., 2024), and this is often interpreted as a consequence of podocyte detachment from the GBM (Kriz et al., 2013; Kriz & Lemley, 2017). However, nephrin ectodomains can be shed from the podocyte cell surface by the actions of metalloproteinases such as MMP‐7, which are markedly elevated in glomerular diseases (Tan et al., 2019) and which can be induced by NFAT signaling cascades in other cell types (Tie et al., 2013; Wang et al., 2015). The results of the present study suggest that continuous TRPC6 activation for 24 h induces ectodomain cleavage by metalloproteinases, and that this pathway requires activation of calcineurin. TRPC6 activation for 24 h induced a calcineurin‐dependent increase in MMP‐7, but this does not exclude a role for other proteases, as Batimistat can inhibit proteases in both the MMP and ADAM families. This raises the possibility that ectodomain shedding contributes to nephrinuria in kidney disease. Note that the overall abundance of podocin in the cell layer was unchanged following 24‐h activation of TRPC6, in marked contrast to what is observed following 24 h exposure to sera from patients with recurrent nephrotic syndromes (Kim et al., 2017). This would not be expected if the soluble nephrin signal observed here was derived from detached cells. In addition, occludin, an essential component of occluding junctions (Shono et al., 2007), was markedly increased under those conditions. Recall that increases in occluding junctions are a feature of podocyte foot process effacement (Kriz et al., 2013; Kriz & Lemley, 2017; Shono et al., 2007; Succar et al., 2016). TRPC6 activation for 24 h did not increase albumin permeability, and it is possible that increases in occludin can compensate for the loss of nephrin ectodomains for a certain period of time. Finally, we observed that while continuous TRPC6 activation for 72 h induces calcineurin‐dependent apoptosis, as described in several reports (Zhang et al., 2009), it is notable that this treatment did not appear to induce pyroptosis.

A weakness of the experimental designs used here is that the system is somewhat artificial. Beyond the obvious fact that the experiments were carried out in vitro on an immortalized podocyte cell line, we also note that TRPC6 activation in the present experiments was continuous, whereas the activation of TRPC6 in vivo is probably periodic. Periodic activation of TRPC6, even if increased during disease states, may be the reason why gain‐of‐function mutations, or increases in cell surface TRPC6 expression, take years to induce a loss of podocyte sufficient to induce overt disease (Heeringa et al., 2009; Reiser et al., 2005; Winn et al., 2005). A strength of the approach used here is that PPZ2 avoids possible activation of parallel non‐TRPC6 pathways that could be engaged by various receptors, including the GPCRs activated by angiotensin II or ATP, or the pattern sensing receptors activated by lipopolysaccharide, which produce quite pleiotropic effects. It also avoids indirect effects mediated by factors secreted by other cell types in glomeruli or elsewhere, which might occur when TRPC6 is activated by administration of agonists in vivo. The fact that all of the positive effects of PPZ2 seen here were antagonized by SAR‐7334 provides additional assurance as to the specificity of the effects observed.

In summary, we have observed that modulation of albumin flux across a confluent layer of immortalized podocytes can occur in as little as 1 h with certain GPCR agonists (e.g., following activation of FPRs), but that TRPC6 activation for 1–24 h does not increase albumin flux. However, TRPC6 activation for 24 h induces nephrin shedding from the cell surface through processes that require activation of calcineurin and metalloproteinases, and this effect is accompanied by an increase in the expression of occludin and MMP‐7. Finally, continuous TRPC6 activation for 72 h induces apoptotic cell death. These data are consistent with a role for podocyte TRPC6 channels in driving the process of foot process effacement, which may play a protective role for certain periods of time during which podocytes are subjected to metabolic, mechanical, or pro‐inflammatory stresses. Finally, while protein permeation across podocytes can be modulated quite rapidly by certain signals, we found no evidence that TRPC6 channels contribute to these processes.

## AUTHOR CONTRIBUTIONS

EYK carried out experiments, analyzed data, and reviewed and edited text and figures. SED acquired grant support, conceptualized the study, wrote initial drafts of the manuscript, and edited text and figures.

## FUNDING INFORMATION

Supported by NIH Grant 5‐R01‐DK104708.

## CONFLICT OF INTEREST STATEMENT

The authors do not have any conflicts of interest, financial or otherwise, to disclose.

## ETHICS STATEMENT

This study does not report experiments carried out on animals, human subjects, or human fetal tissue.

## Supporting information


Figure S1.

## Data Availability

Data will be made available upon reasonable request.
