# Supplementary material for: TRPC6 effects on albumin permeation, nephrin shedding, and apoptosis in podocytes: Role of calcineurin and metalloproteases
Source: Physiol Rep. 2025 Oct 30;13(21):e70614. doi: 10.14814/phy2.70614 (PMC12573276; doi:10.14814/phy2.70614)
Supplement: Supplementary file 1 — Figure S1. [file PHY2-13-e70614-s001.pdf]

A

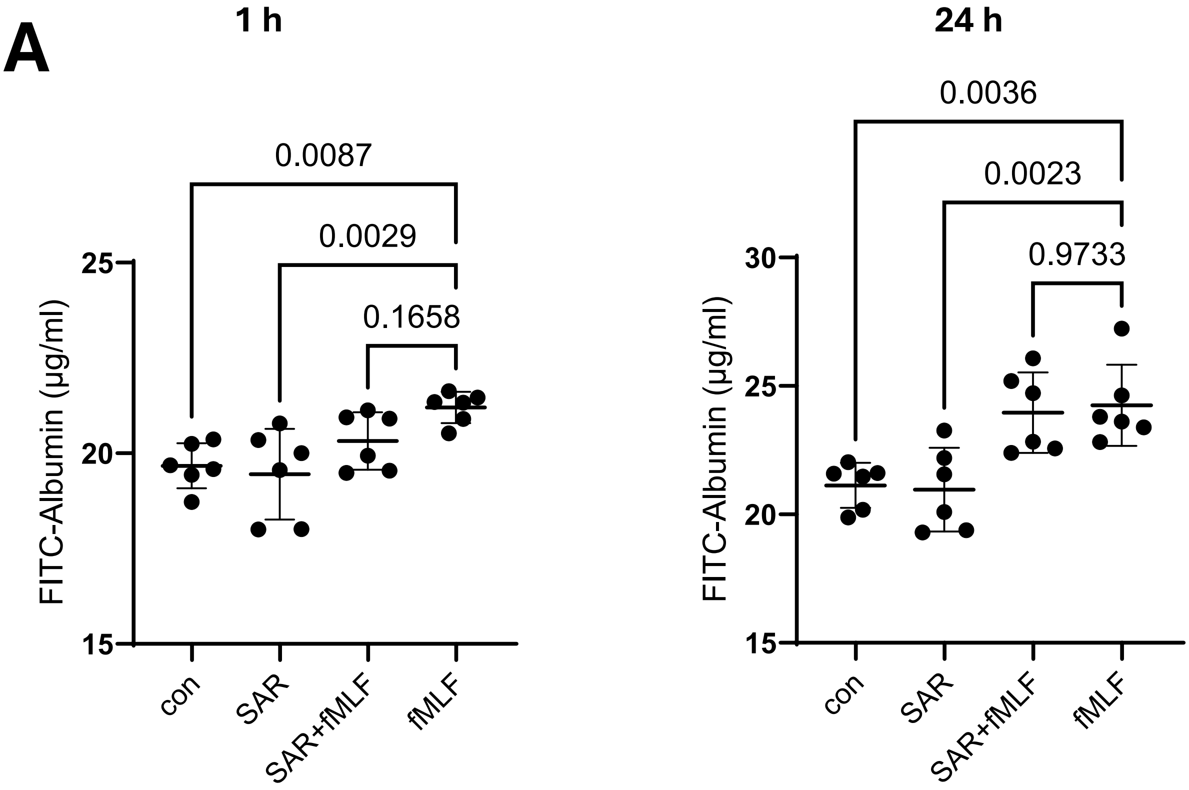

The TRPC6 blocker SAR-7334 does not inhibit increases in albumin permeation evoked by the FPR agonist fMLF for 1 h or 24 h.

B

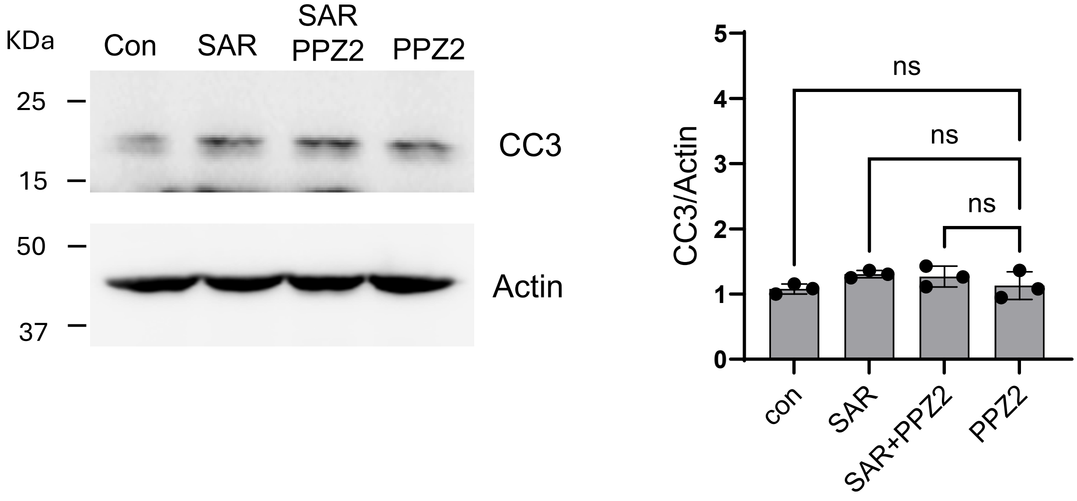

TRPC6 activation for 24 h is not sufficient to evoke apoptosis in podocytes

Supplemental data
